# Anesthesia

**Published:** 1852-07

**Authors:** 


*Anesthesia.*—The British and Foreign Medico-Chirurgical Review, for January, gives the following summary of the deaths from anesthesia, in the order of the occurrence of the cases. The list does not include several cases which have occurred within the last eighteen months, nor, as the writer remarks, "at least five well-authenticated deaths from needless and careless use of chloroform in this country (England) alone, or of numerous cases where dangerous symptoms have been observed, or death obscurely traced:

"1. Hannah Greener, aged fifteen, was greatly afraid of respiring the chloroform,—only about two drachms were used,—insensibility was produced in half a minute, when the removal of a great toe-nail was commenced; death occurred in about two minutes.

2. Case at Cincinnati, aged thirty-five, apparently in good health; was chloroformed for about a minute for extraction of a tooth; death occurred in from five to ten minutes.

3. Patrick Coyle, chloroformed for fistula; he inhaled for about a minute, and almost instantly expired.*

4. Mdlle. Stock, Boulogne, aged thirty, subject to palpitation, and chlorotic; inhaled about fifteen or twenty drops; operation, opening of an abscess in hip; death almost instantaneous.

5. Daniel Schlyg, aged twenty-four, had a thigh fractured by a ball during the days of June, in Paris, and was in a state of profound depression; he inhaled the chloroform for about three minutes, amputation at the hip-joint was performed, and death occurred in three quarters of an hour.

6. Walter Badger, aged twenty-three, had heart and liver disease; he inhaled chloroform for about a minute, previous to the extraction of a tooth; the operator, Mr. Robinson, of London, was absent for less than a minute to seek more chloroform, and found his patient dead on his return. The inhalation took place from an apparatus.

7. A young woman of Hyderabad was chloroformed for amputation of middle finger of left hand; about a drachm of chloroform was used; death almost instantaneous.

8. John Griffith (Warren) had chancres and hemorrhoids; inhaled about three drachms, and died in about ten minutes, during the excision of the hemorrhoids.

9. Abbey Pennock (Warren) inhaled about three drachms in two applications, to relieve the pain of toothache, and died almost immediately after the second application.

---

*The case of Coyle is given by Dr. Warren alone.

tion, subdues inflammation, and most frequently induces a sleep, which becomes truly the crisis of the disease."—*Ibid.*

10. Example by M. Malgaigne. A man, one of the wounded of the three days of June, had the humerus broken by a ball, and was a good deal weakened by hemorrhage and gangrene of the wound; was chloroformed, and the humerus disarticulated; new inhalations were taken during the search after the ball, and he died during the last incision.*

11. Charles Denoyers, aged twenty-two, was affected with white swellings of the left wrist, and chloroformed at the Hotel Dieu, of Lyons, for five minutes before and during cauterization of the tumours; death took place shortly after the commencement of the operation.

12. Case of M. Roux; removal of a scirrhous breast; death before quitting the amphitheatre.

13. Case described by M. Guérin, as having taken place at the Bicêtre. A man affected with a lesion of the thigh was chloroformed for amputation at the hip-joint; death took place before the end of the operation.

14. Case at Govan; a young man chloroformed for removal of a great toe-nail; death almost immediate.

15. Case of M. Barrier, of Lyons; a young man, named Verrier, aged seventeen, inhaled from six to eight *grammes* of chloroform for about six minutes, for an amputation of the finger, and died in about half a minute afterwards.

16. At the Hospital of Madrid, (Bouisson,) a child of twelve years being chloroformed for an amputation of the leg, was seized with a tetanic spasm, and died in a minute and a half.

17. By M. de Confevron, of Langres. Madame Labrune, aged thirty-three, of a nervous temperament, was chloroformed for the extraction of a tooth; almost instant death.

---

*Dr. Warren gives 10 fatal cases, but inserts the cases which we have omitted for the reasons above given. This last case is our 9th; and our 11th is the 7th of M. Bouisson, who, like us, excludes suicidal cases, and such like. He gives 15 cases; his last is our 17th.

18. Mr. Solly's case; a man, aged forty-eight, apparently in perfect health, inhaled rather more than a drachm of chloroform from an inhaler for about three minutes; then a toe-nail was removed; and death took place in about six or seven minutes from the beginning.

19. Case at Leeds, Robert Mitchell; chloroform applied during an attack of delirium tremens by Mr. Teale; death about an hour afterwards. (Hardly a clear case.)

20. Case at Shrewsbury; a girl named Jones; only a small quantity of chloroform was given before proposed extirpation of the eyeball; almost instant death, as if from apoplexy.

21. Case at Berlin; a young lady died during the extraction of a tooth, from the alleged effects of about half a drachm of chloroform.

22. Case at Guy's Hospital, referred to in 'Med. Gaz.' for June, 1850.

23. Aschendorf's case; a child of one year old operated on for a nævus under the influence of chloroform; only about nine drops were used; death on removal from the table.—*British & Foreign Medico-Chirurgical Review.*

